# Genetic Diversity and Breeding Strategies for Resistance to Yellow Rust (*Puccinia striiformis* f. sp. *tritici*) in Wheat Hybrid Populations Based on Phenotypic and DNA Marker Screening

**DOI:** 10.3390/plants15131964

**Published:** 2026-06-25

**Authors:** Saltanat Dubekova, Shynar Mazkirat, Dilyara Babissekova, Sholpan Khalbaeva, Amangeldy Sarbayev, Shynbolat Rsaliyev, Isatay Nurpeisov, Aydarkhan Yesserkenov

**Affiliations:** 1 Laboratory of Immunity and Plant Protection, Kazakh Research Institute of Agriculture and Plant Growing, Almaty District, Almalybak 040909, Kazakhstan; shynbolat63@mail.ru (S.R.); ajs-eserkenov@mail.ru (A.Y.); 2 Laboratory of Molecular Plant Biology, Kazakh Research Institute of Agriculture and Plant Growing, Almaty District, Almalybak 040909, Kazakhstan; shynarbek.mazkirat@gmail.com (S.M.); dilyara280188@mail.ru (D.B.); sholpan_2706@mail.ru (S.K.); 3 Laboratory of Grain Crops, Kazakh Research Institute of Agriculture and Plant Growing, Almaty District, Almalybak 040909, Kazakhstan; nisatay@mail.ru

**Keywords:** wheat, yellow rust, breeding, resistance, lines, molecular markers, *Yr* genes

## Abstract

Yellow rust (*Puccinia striiformis* f. sp. *tritici*) is one of the most destructive diseases in wheat (*Triticum aestivum* L.) in Kazakhstan, causing significant yield losses. Owing to the high susceptibility of widely cultivated varieties, the development of resistant genotypes remains a key objective for sustainable crop protection. The aim of this study was to evaluate the resistance of wheat lines to yellow rust and to identify effective resistance genes. The research was conducted under artificial infection conditions using hybrid populations of the F2–F5 generations. The genotypes were assessed and ranked according to their resistance levels, and molecular markers were applied to detect resistance genes. Significant variability in disease response was observed. Analysis of variance revealed a strong effect of genotype on the infection coefficient (*p* < 0.001). Lines from later generations (F5) presented lower infection levels. Most genotypes carried the *Yr5* gene, highlighting its major role in resistance, whereas *Yr10* was less common. *Yr15* and *Yr18* were detected in some lines and were associated with partial (adult plant) resistance. Moderately susceptible forms predominated, indicating widespread quantitative resistance. However, highly resistant lines (CI = 0–1) and immune forms were identified, representing valuable material for breeding programs.

## 1. Introduction

Among the most important food crops worldwide is wheat (*Triticum aestivum* L.), which plays a key role in ensuring global food security. It accounts for a significant share of global grain production, and the resistance of wheat crops to biotic stresses directly affects yield stability. Among wheat diseases, rust diseases, including yellow rust caused by the obligate biotrophic fungus *Puccinia striiformis* f. sp. *tritici*, are particularly dangerous. In recent decades, the harmfulness of yellow rust has increased significantly in many regions of the world, including Europe, Asia, North America, and Russia. This trend is associated with global climate change, active pathogen migration, and the emergence of new highly virulent races [1,2,3,4,5].

Yellow rust can cause substantial yield losses, reaching 30–70% during epidemic years, and, in some cases, can lead to almost complete crop failure in susceptible varieties. The disease develops most intensively under conditions of moderate temperatures and high humidity, making it particularly dangerous in temperate wheat-growing regions [6].

Although chemical control methods are effective, they are associated with economic costs and environmental risks and contribute to the development of pathogen resistance to fungicides. Therefore, the breeding and deployment of resistant varieties are considered the most environmentally sustainable and economically viable strategies for disease control [7,8].

The genetic resistance of wheat to *Puccinia striiformis* is characterized by considerable diversity. To date, more than 80 officially designated yellow rust resistance genes (*Yrs*) have been identified, along with dozens of quantitative trait loci (QTLs) involved in resistance expression at different stages of plant development [9]. On the basis of their expression, resistance is classified into all-stage resistance (ASR), which is typically race-specific and controlled by one or several major genes, and adult plant resistance (APR), which is generally partial but more durable [10].

Most *Yr* genes identified thus far belong to the ASR group and are highly effective against specific pathogen races. However, their widespread use in breeding has repeatedly led to resistance breakdown due to the rapid evolution of *P. striiformis* populations and the emergence of new virulent races [11,12]. In many regions worldwide, the effectiveness of previously widely used genes such as *Yr6*, *Yr7*, *Yr9*, *Yr17*, and *Yr27* has been lost [4,13]. This highlights the need to identify new resistance sources and develop strategies to increase durability.

In recent years, particular attention has been given to broad-spectrum resistance genes, including *Yr15*, *Yr39*, and *Yr62*, as well as APR genes, such as *Yr18*, which are characterized by race nonspecificity and slow disease development [14,15,16]. The *Yr15* gene, introgressed from wild emmer wheat (*Triticum dicoccoides*), is considered one of the most promising sources of resistance; however, its presence in cultivated varieties remains limited [17]. Similarly, the introgression of resistance genes from wild and related wheat species has increased the genetic diversity of breeding material but requires precise molecular identification of introgressed chromosomal segments [18]. The genes *Yr5*, *Yr10*, *Yr15*, and *Yr18* were used in the present study because they are considered among the most effective and widely studied sources of resistance to yellow rust in wheat. *Yr5*, *Yr10*, and *Yr15* are major race-specific resistance genes that provide high levels of resistance against diverse pathotypes of *Puccinia striiformis* f. sp. *tritici*, whereas *Yr18* is associated with partial adult plant resistance and durable field resistance. The combination of major and adult plant resistance genes is considered an effective strategy for achieving stable and long-term protection against yellow rust. Therefore, these genes are widely used in wheat breeding programs and represent important targets for marker-assisted selection and gene pyramiding approaches.

Modern approaches to studying wheat resistance to yellow rust are based on the integration of phytopathological analysis with molecular genetic methods. The use of DNA markers linked to *Yr* genes and QTLs enables faster identification of resistance genes, increases selection accuracy, and facilitates effective gene pyramiding in breeding programs [19,20,21]. These approaches are widely applied both in the analysis of hybrid populations and in the evaluation of cultivars included in national breeding registries of different countries [2,5,22].

Monitoring the resistance of advanced wheat cultivars is particularly important, as they form the basis of agricultural production. Studies conducted in Russia, China, Central Asian countries, and Europe indicate that many modern cultivars contain a limited set of *Yr* genes, often represented by translocations of 1BL.1RS or 1AL.1RS carrying *Yr9* and *Yr17*, whose effectiveness has significantly declined in recent years [2,13,23]. Moreover, combining such genes with APR and other resistance sources may increase the overall level of resistance and increase the genetic diversity of cultivars.

Given the ongoing evolution of *Puccinia striiformis* populations, characterized by high genetic plasticity and long-distance migration ability, an integrated assessment of wheat resistance considering both phenotypic and molecular data is particularly relevant [24,25]. The identification of resistant genotypes and analysis of their genetic basis are essential prerequisites for developing effective breeding strategies aimed at producing cultivars with durable and stable resistance to yellow rust.

The novelty of the present study lies in the expansion of wheat genetic diversity through the identification of promising lines with resistance to yellow rust on the basis of the integration of phenotypic screening and DNA marker analysis of *Yr* genes. This integrated approach will provide a comprehensive understanding of the genetic diversity of resistance and the effectiveness of breeding strategies in national wheat improvement programs, as well as identify valuable and promising genotypes for subsequent inclusion in sustainable variety development programs.

The aim of this study was to increase the genetic diversity of wheat with resistance to yellow rust through comprehensive analysis of phenotypic responses and DNA marker-based screening of *Yr* genes.

## 2. Results

### 2.1. Meteorological Conditions During the Study Period

The climatic conditions during the growing season of winter wheat from 2021 to 2025 were characterized by considerable interannual variability in air temperature and precipitation (Table 1).

The mean monthly air temperature during the growing season ranged from 17.2 °C (2021) to 22.4 °C (2025), with the highest monthly temperatures observed from June–July (up to 27.7 °C). Precipitation varied from 37.3 mm (2023) to 84.3 mm (2024). The driest conditions were recorded in 2023, particularly in June (4.3 mm), whereas 2024 was characterized by greater moisture availability. Weather conditions in 2025 are characterized by a combination of high temperatures and precipitation deficits, creating stress conditions for plants. Overall, the study years differed significantly in terms of hydrothermal conditions, which influenced plant growth, development, and the progression of phytopathogens.

### 2.2. Phenotypic Screening of Wheat Line Resistance to a Yellow Rust Population (Puccinia striiformis f. sp. tritici)

**Figure 1:**
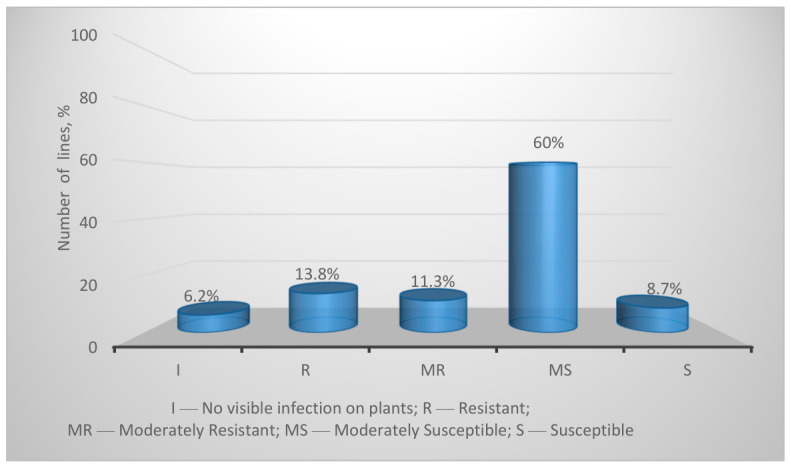
Distribution of wheat lines according to resistance to yellow rust (*Puccinia striiformis* f. sp. *tritici*), 2024.

During the study period, commercial cultivars such as Zhetysu, Almaly, Bogarnaya 56, and Steklovidnaya 24 were infected by the pathogen at levels ranging from 30 to 60%, with a reaction type ranging from MS to S, whereas the foreign indicator cultivar Morocco was infected up to 100S. Analysis of the distribution of hybrid population lines according to their resistance to yellow rust (*Puccinia striiformis* f. sp. *tritici*) revealed that the largest proportion of the hybrid population lines was in the moderately susceptible (MS) category, accounting for 60% of the total. This indicates the predominance of genotypes with an intermediate level of plant infection (Figure 1).

Lines characterized by resistance (R) accounted for 13.8%, whereas the proportion of moderately resistant (MR) forms was 11.3%. Thus, the combined share of resistant and moderately resistant lines did not exceed 25.1%. The susceptible (S) lines represented 8.7% of the total, indicating the presence of highly sensitive genotypes. The lowest proportion was observed in the group showing no visible symptoms of infection (I), which accounted for 6.2%, reflecting the rare occurrence of complete resistance. Most lines were characterized by moderate susceptibility to yellow rust (*Puccinia striiformis* f. sp. *tritici*), whereas fully resistant or completely healthy forms were present in limited numbers. This highlights the need for further breeding efforts to increase resistance (Supplementary Table S1).

In 2024, favorable conditions for disease development were observed, resulting in a high infection background that enabled a comprehensive evaluation of the resistance of the studied material. Analysis of the infection coefficient revealed significant variability in disease severity among lines. The infection levels of the F2–F5 hybrid population lines ranged from zero to high values (up to 24–50%), indicating the presence of both highly resistant and susceptible genotypes (Table 2).

For the F2 hybrid generation, the mean value was 21.2%, with a median of 20.0%, indicating a moderate level of infection in most lines. The wide range of values (0–50%) reflects substantial genotypic differentiation in resistance. A similar trend was observed for F3, where the mean value (11.8%) was slightly lower than the median (12.0%), suggesting a predominance of lines with relatively low infection levels, despite the presence of a few highly infected genotypes. For F4, the mean value (13.43%) exceeded the median (12.0%), which may indicate the influence of several genotypes with relatively high infection levels on the overall distribution. The variation range (0–50%) also confirms the high heterogeneity of the studied material. The lowest infection coefficient values were recorded for F5 (mean of 7.95%, median of 4.0%), indicating greater resistance in most lines for this trait. However, the maximum values reaching 24% suggest the presence of susceptible forms within this group.

Overall, the results demonstrate pronounced genetic heterogeneity among wheat lines in terms of resistance to yellow rust (*Puccinia striiformis* f. sp. *tritici*), enabling the identification of promising resistance sources for further breeding.

Analysis of variance (ANOVA) of the infection coefficient (CI) revealed a statistically significant effect of the hybrid factor on disease severity. The differences among the F2–F5 hybrid population lines were significant (F = 6.17; *p* = 0.000823) (Table 3).

The high statistical significance (*p* < 0.001) indicates substantial differentiation among lines in terms of the infection coefficient. This makes it possible to identify genotypes with varying levels of disease resistance and confirms their potential for use in breeding programs.

For a more detailed analysis of differences between lines, Tukey’s HSD test was applied. Statistically significant differences were observed only between the F2 and F5 generations (*p* = 0.0006), with the infection coefficient in F5 being significantly lower than that in F2. Differences between the other pairs of the hybrid population (F2–F3, F2–F4, F3–F4, F3–F5, and F4–F5) were not statistically significant (*p* > 0.05), despite a general trend toward a reduction in infection levels (Table 4).

Thus, the results of the multiple comparisons confirmed that the greatest reduction in the infection coefficient was observed in the F5 generation compared with the F2 generation, whereas the remaining generations formed statistically similar groups. Overall, this indicates a gradual increase in resistance during the breeding process and allows the F5 lines to be considered the most promising in terms of resistance to infection.

**Figure 2:**
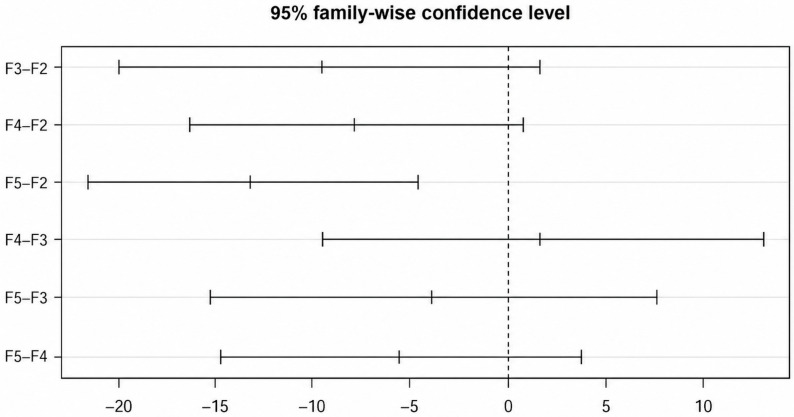
Differences in mean levels of coefficient infection among hybrid lines of populations F2–F5.

The graph presents 95% confidence intervals for the differences in the mean infection coefficient values among the hybrids. Visualization confirmed that the F5 generation presented the highest level of resistance, whereas the other generations formed statistically similar groups with respect to the infection coefficient (Figure 2).

**Figure 3:**
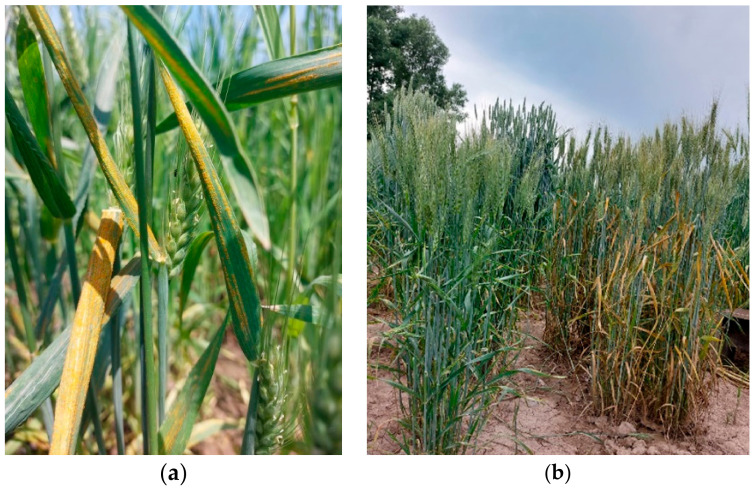
Phenotypic differentiation of wheat genotypes under yellow rust infection caused by *Puccinia striiformis* at the adult plant stage. June 2024: (**a**) Representative infection symptoms; (**b**) Differential response of wheat genotypes under controlled artificial infection with a population of *Puccinia striiformis*.

Thus, the evaluation of resistance in wheat hybrid population lines under conditions of intensive yellow rust development in the region, together with the analysis of their immunological potential, made it possible to eliminate several susceptible accessions and select promising lines at the experimental station (Figure 3). The parental material of the studied lines included locally adapted high-yielding cultivars with valuable agronomic traits, which increases the breeding value and practical significance of the selected resistant lines. As a result of selecting resistant lines from the total studied material, a set of immunologically valuable wheat forms resistant to the yellow rust population (*Puccinia striiformis* f. sp. *tritici*) was developed.

### 2.3. Molecular Genotyping of Effective Yr Resistance Genes

**Figure 4:**
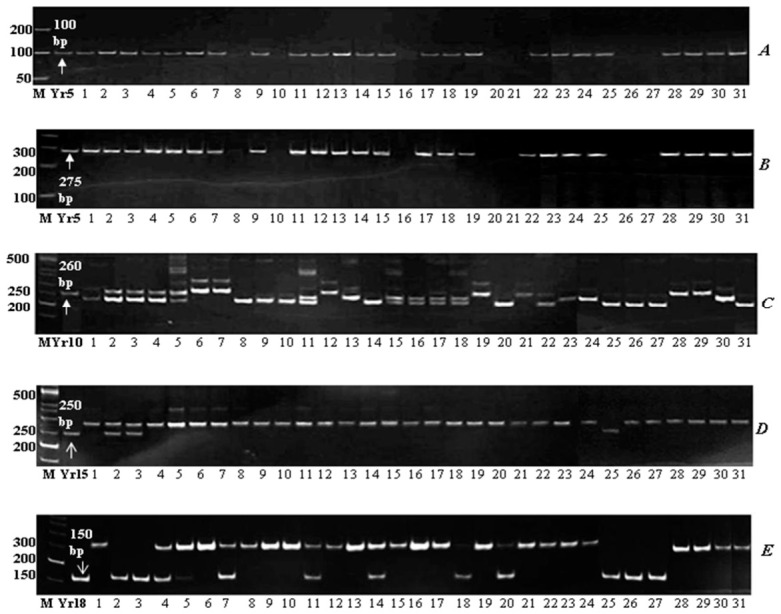
DNA amplification products of wheat line samples linked to the *Yr5*, *Yr10*, *Yr15*, and *Yr18* genes. (**A**,**B**) *Yr5* identification via the STS markers S19M93-100 and S23M41-275, respectively. Band presence indicates the *Yr5* resistance gene; (**C**–**E**) represent the identification of *Yr10*, *Yr15* and *Yr18*, respectively.

The genotyping of advanced wheat lines has made it possible to identify the presence of effective resistance genes. Analysis of the occurrence of *Yr* genes in the studied lines revealed their different distributions and contributions to the development of resistance to yellow rust (Figure 4).

The *Yr5* gene is present in most of the studied lines and is among the most widely distributed genes, likely making a major contribution to resistance expression. Lines carrying *Yr5* in some cases presented high and moderate levels of resistance (R–MR) with low infection coefficient values (CI = 0–8), confirming its effectiveness under the conditions of 2024. The *Yr10* gene was less common; however, in combination with *Yr5* (in certain hybrid combinations), it contributed to high resistance (R, CI = 1), indicating a positive effect of gene pyramiding.

Although *Yr15* is known for its high effectiveness, in the studied material, it was often detected in a heterozygous state or was absent and did not always confer a high level of resistance. This may indicate its incomplete expression in the tested genotypes or the influence of genetic background. The *Yr18* gene was identified in several lines, mainly in a heterozygous state, and was associated with moderate resistance (MR–MS). Its effect is likely partial, and it is expressed in combination with other genes. The most valuable breeding lines were those with gene combinations (*Yr5* + *Yr10*, *Yr5* + *Yr18*), which provided stable resistance, whereas single genes or their absence resulted in reduced plant protection. The advanced wheat lines analyzed via molecular markers are presented in Supplementary Table S2.

Thus, the hybrid population lines were evaluated for their immunological characteristics, and genetic-level information was obtained regarding their effectiveness and resistance to *Puccinia striiformis* f. sp. *tritici.* Promising hybrid combinations were identified. As an output of the study, valuable lines carrying *Yr* genes were obtained, confirming the effectiveness of yellow rust resistance under field conditions.

These results are consistent with the current understanding of the genetic basis of wheat resistance to yellow rust and confirm the appropriateness of selecting lines with resistant and moderately resistant reactions as donors of durable resistance traits.

### 2.4. Selection of Promising Wheat Lines Resistant to Yellow Rust (Puccinia striiformis f. sp. tritici)

Heatmaps and clustering are important analytical tools for visualizing patterns, similarities, and underlying structures in complex datasets, thereby facilitating data interpretation and exploratory analysis. Molecular marker data were scored as binary values, where “0” indicated absence and “1” indicated the presence of the target marker or gene. These binary data were used to generate a heatmap for visualizing genotype–marker associations. A two-color scheme was applied to represent the absence and presence of markers, which is standard for binary genotype data visualization. In this study, both heatmaps and hierarchical cluster analysis were performed by including isogenic control lines carrying *Yr5*, *Yr10*, *Yr15*, and *Yr18*. These control genotypes were used as references to evaluate the genetic relationships between the breeding lines and known sources of yellow rust resistance.

Hierarchical cluster analysis of genotypes, combined with data on the molecular identification of resistance genes (*Yr5*, *Yr10*, *Yr15*, and *Yr18*) and the coefficient of infection (CI), made it possible to further clarify the structure of resistance to yellow rust among the studied samples.

**Figure 5:**
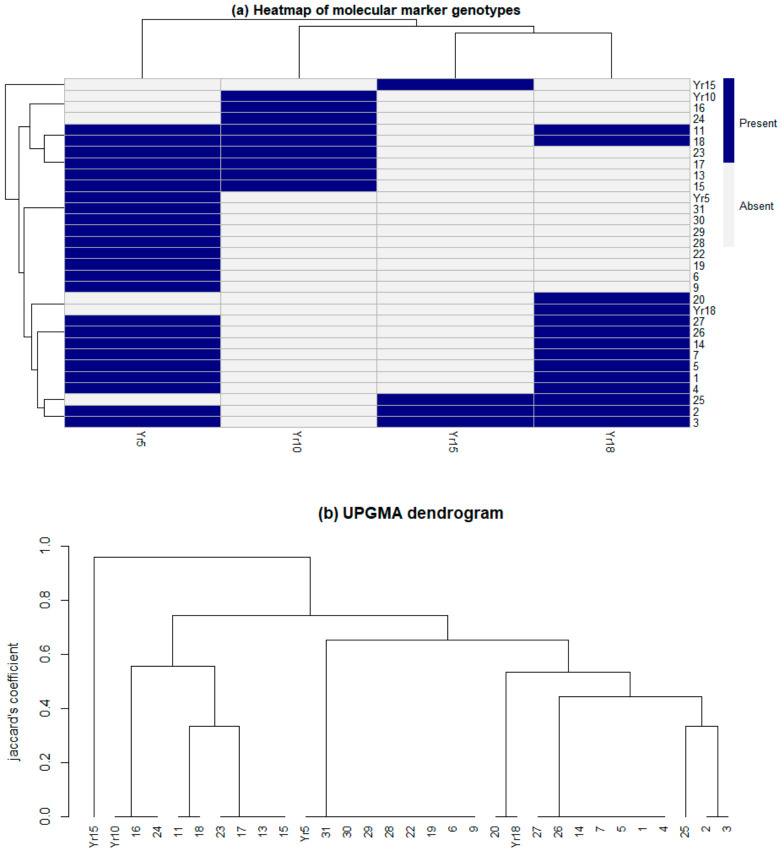
Heatmap and UPGMA clustering of wheat genotypes (breeding lines and isogenic controls) on the basis of molecular marker profiles associated with yellow rust resistance. (**a**) Heatmap of molecular marker genotypes. The X-axis represents the presence/absence of *Yr* genes (*Yr5*, *Yr10*, *Yr15*, and *Yr18*); on the Y-axis, numbers 1–31 represent the studied lines, whereas *Yr5*, *Yr10*, *Yr15* and *Yr18* represent isogenic lines. (**b**) UPGMA dendrogram constructed using the Jaccard distance to show genetic relationships among genotypes. On the x-axis, numbers 1–31 represent the studied lines, whereas *Yr5*, *Yr10*, *Yr15*, and *Yr18* represent isogenic lines.

On the basis of the dendrogram, several clusters were identified that differed in the level of resistance. Within individual clusters, genotypes with similar phenotypic responses to the disease were grouped together, indicating a possible shared genetic basis of resistance (Figure 5).

Genotypes predominantly carrying the *Yr5* and *Yr10* genes formed compact clusters (lines 24, 11, 18, 23, 17, 13, and 15). Lines numbered 30, 29, 22, 6, 28, and 19 formed a separate cluster, whereas lines showing variable reactions comprised genotypes 5, 1, 4, 2, and 3. Susceptible forms (CI ≥ 20) were separated into distinct subgroups. Several clustered genotypes were characterized by low coefficients of infection (CI = 0–1) and resistance reactions (I–R), suggesting a possible association between genetic similarity and yellow rust resistance.

Genetically, the most closely related lines forming tight subclusters (1–4, 2–3; 28–22, 29–30; 24–11–18–23–17–13–15) were characterized by minimal dendrogram distances. This finding suggests their probable close genetic origin and similar combinations of resistance genes, primarily *Yr5*.

The analysis revealed that high resistance to yellow rust (R, CI = 0–1) was characteristic of a limited number of lines, among which the most valuable for breeding was d.1010 (d.93 F3 (N 23 × Kupava) × Mereke × 10/60 F5 N23 Kupava 7 (CI = 1), Alpu/VR5053 (WA#FM/201/232/GS50A) × Steklovidnaya 24 (CI = 1), Yr5/6 Avocet S × 20389-6 (CI = 1), F5 №23 × Kupava/5 × 37/20948-8 (CI = 1), MV-Menuett × 13/d 3 gene (CI = 1), SG-V9157 × 22/20060-3 (CI = 1), and F5 №23 × Kupava/10 × Mamyr (CI = 1). These genotypes generally carry the *Yr5* gene alone or in combination with other genes (e.g., *Yr10*), confirming its key role in resistance expression.

Complete resistance (I, CI = 0) was observed in the following lines: Yr5/6* Avocet S × 16/12 × Triticum spelta; F5 №23 × Kupava/10 × 35/20060-2; Yr15/6* Avocet S × 20389-6; CH-111.14098 × OR2080111H; Yr15/6* Avocet S × Sultan. Despite the absence of infection, some of these genotypes have not yet been fully characterized at the genetic level for all effective resistance genes, indicating the need for further investigation of their genetic background.

The group of moderately resistant forms (MR, CI = 4–8) included lines 4/2109 × Yr5/6* Avocet S (CI = 4), F5 №23 × Kupava/1 × 48/12121-6 (CI = 6), and SO1-249-14*R × 57/21190-1 (CI = 8). These genotypes mainly carry *Yr5*, sometimes in combination with *Yr18* (in a heterozygous state), indicating partial resistance expression.

Thus, analysis combined with molecular identification of resistance genes and coefficient of infection (CI) assessment allowed the identification of resistant genotypes (CI = 0–8) carrying *Yr5* and *Yr10*; moderately resistant groups with limited gene effectiveness; susceptible forms (CI ≥ 20) subject to elimination; and potential donors representing new sources of resistance to yellow rust populations for expanding wheat genetic diversity.

## 3. Discussion

The present study demonstrated substantial variability in resistance to yellow rust among the evaluated F2–F5 winter wheat lines under artificial infection conditions. The obtained results made it possible to identify resistant genotypes carrying effective *Yr* genes, particularly *Yr5* in combination with *Yr10* and *Yr18*, which represent valuable sources of resistance for wheat breeding programs.

The evaluation of wheat lines from the F2–F5 generations for resistance to yellow rust (*Puccinia striiformis* f. sp. *tritici*) under artificial infection conditions revealed significant differences among genotypes in their response to the pathogen. The predominance of lines classified as moderately susceptible (MS, 60%) indicates the widespread occurrence of partial, quantitative resistance to the pathogen. Similar results have been reported in studies showing that most breeding populations are dominated by genotypes with intermediate reactions to the pathogen, which is associated with polygenic trait control and strong environmental influence [26,27].

The relatively low proportion of resistant (R, 13.8%) and moderately resistant (MR, 11.3%) lines is consistent with reports on the reduced effectiveness of vertical resistance to yellow rust under conditions of high pathogen variability. Resistance controlled by individual *Yr* genes is often overcome by the emergence of new virulent races of *P. striiformis* [26,28]. In this context, horizontal resistance, which slows disease development while maintaining plant productivity, is becoming increasingly important.

The presence of susceptible lines (S, 8.7%) confirms the genetic heterogeneity of the studied material and is consistent with field observations, indicating that even modern cultivars and breeding lines may exhibit high levels of disease under favorable environmental conditions for yellow rust pathotypes [29]. This highlights the need for continuous monitoring of resistance and regular updating of breeding material. The small proportion of symptomless forms (I, 6.2%) indicates the rarity of highly resistant sources. Studies suggest that such genotypes are often associated with combinations of multiple QTLs or specific *Yr* genes and represent valuable material for breeding programs [30,31].

The analysis of the coefficient of infection in the studied lines in 2024 under high disease pressure revealed substantial variability. The values ranged widely from 0 to 24–50%, indicating the presence of both resistant and susceptible genotypes. Overall, most lines presented a moderate level of infection; however, individual combinations presented both minimal and high infection coefficients, which is consistent with the polygenic nature of yellow rust resistance and its dependence on genotype–environment interactions [32,33].

The obtained data are supported by the results of analysis of variance. The hybrid factor had a significant effect on the coefficient of infection (F = 6.17; *p* = 0.000823), indicating that the variability observed in Table 4 was largely determined by genetic differences among the studied lines. The statistically significant effect of the hybrid factor on the infection coefficient (*p* < 0.001) further confirms the genetic basis of the observed differences. This finding is consistent with previous studies showing that variability in resistance in segregating populations is determined by combinations of resistance genes and their interactions [34].

Multiple comparison (HSD) results revealed statistically significant differences between the F2 and F5 generations, with F5 lines exhibiting lower infection levels. This may indicate the accumulation of favorable resistance alleles during the selection process, as also reported in studies on cereal crop improvement [35]. The differentiation of the studied wheat lines from the F2–F5 generations in terms of resistance in 2024 allows the identification of the most promising genotypes for further breeding work. This enables the reliable selection of resistant groups and the justification of breeding material selection.

In breeding for rust resistance, the incorporation and selection of resistance sources can be implemented and is feasible at early generations, which is consistent with modern approaches to accelerated wheat breeding [35]. Early identification of resistant forms makes it possible to significantly reduce the scope of subsequent testing and improve selection efficiency. Sources of durable resistance represent more complex genotypes that involve the introgression of multiple genes without affecting other agronomically important traits in the final product. Unlike monogenic resistance, such resistance provides broader and more stable protection to plants without negatively influencing economically valuable traits [33].

In this context, gene combinations conferring both all-stage resistance (ASR) and adult plant resistance (APR) are of particular value. The methodology applied in the present study, including hybridization (pairwise and backcrossing schemes), enabled effective introgression of *Yr* resistance genes into the genetic background of the studied lines. The use of DNA markers to confirm the presence of resistance genes significantly increased selection accuracy and allowed monitoring of the introgression process at the molecular level, which is a key component of marker-assisted selection (MAS) [32].

The identified lines carrying individual resistance genes represent valuable initial material for further gene pyramiding. The combination of ASR and APR genes, as well as minor resistance genes, contributes to the development of long-term and broad-spectrum resistance to different pathotypes of *Puccinia striiformis* f. sp. *tritici* [35]. Gene pyramiding strategies can be effectively implemented through backcrossing schemes using marker-assisted selection and identified QTLs, allowing resistance to be combined with the preservation of desirable agronomic traits. Such approaches are widely applied in modern breeding programs and are considered among the most promising strategies for developing resistant wheat cultivars [33].

The scientific novelty of this study lies in the integrated approach combining phenotypic screening under high natural and artificial infection pressure with DNA marker-based identification of *Yr* resistance genes in genetically diverse wheat hybrid populations (F2–F5) developed through complex stepwise and backcross breeding schemes. Unlike many previous studies focused mainly on commercial cultivars or single-gene analyses, this work evaluated segregating breeding material derived from long-term selection for yellow rust resistance under local agroecological conditions in southeastern Kazakhstan. The innovation of this study is also reflected in the simultaneous use of the infection coefficient (CI), multiseason field evaluation, molecular detection of key *Yr* genes (*Yr5*, *Yr10*, *Yr15*, and *Yr18*), and cluster analysis to identify stable resistant genotypes and favorable gene combinations. This integrative strategy enabled the identification of novel and regionally adapted breeding lines with high resistance to *Puccinia striiformis* f. sp. *tritici*, providing valuable genetic resources for marker-assisted pyramiding of resistance genes in wheat improvement programs.

Specifically, in the present study, the observed predominance of moderately susceptible genotypes and the relatively low frequency of highly resistant lines are consistent with previously published reports indicating the quantitative and polygenic nature of resistance to *Puccinia striiformis* f. sp. *tritici* in segregating wheat populations. The significant genotypic effect on the infection coefficient (CI), together with the progressive increase in resistance from the F2 to F5 generations, supports the hypothesis that recurrent selection and the accumulation of favorable alleles contribute to increased resistance across successive breeding cycles. Furthermore, the identification of *Yr5* as one of the key resistance determinants, often in combination with *Yr10* and *Yr18*, is consistent with previous studies demonstrating the effectiveness of gene pyramiding in achieving more stable and durable resistance.

The identified resistant genotypes and effective combinations of Yr genes are of particular importance under conditions of high variability in *Puccinia striiformis* populations and the expanding distribution of yellow rust due to climate change. The continuous emergence of new virulent pathogen races has reduced the effectiveness of individual resistance genes, highlighting the need to identify new sources of resistance and develop strategies for durable plant protection. In this context, the use of marker-assisted selection (MAS) combined with field evaluation of resistance significantly improves selection accuracy and accelerates the development of resistant wheat cultivars. Of particular practical importance are lines carrying combinations of *Yr5*, *Yr10*, and *Yr18* genes, since gene pyramiding contributes to broader and more stable resistance against diverse pathogen pathotypes. These results confirm the potential of integrating molecular, genetic and phenotypic approaches to increase the genetic diversity of resistance and improve the adaptability of modern wheat cultivars under changing climatic conditions.

## 4. Materials and Methods

### 4.1. Plant Material

The plant material consisted of a hybrid population (F2–F5) comprising 80 wheat lines (Table 5). The development of promising lines was carried out through hybridization of yellow rust-resistant sources via intraspecific crosses (*Triticum aestivum* L.): pairwise and reciprocal crosses, as well as complex stepwise and backcross (recurrent) crossing schemes [36].

Isogenic lines (*Yr*) of the Avocet cultivar provided by the International Maize and Wheat Improvement Center (CIMMYT, Turkey) were used as parental forms, along with resistant genotypes selected from the Gene Bank collection and high-yielding cultivars, including Egemen 20, Daulet, Mereke 70, Mamyr, Karasai, Almaly, Nureke, Steklovidnaya 24, and Arap, developed through local breeding programs at the Kazakh Research Institute of Agriculture and Plant Growing (KRIAPG).

Commercial cultivars Almaly, Zhetysu, Steklovidnaya 24, and Bogarnaya 56 and the susceptible indicator cultivar Morocco were used as standards. The complete list of the studied hybrid population lines and the origin of the breeding lines are provided in Supplementary Tables S3 and S4, respectively.

### 4.2. Field Experiments and Trials

Research (2020–2025) was conducted in the southeastern region of Kazakhstan at the experimental field of the Kazakh Research Institute of Agriculture and Plant Growing (KRIAPG; 43°13′23″ N, 76°53′57″ E). Seeds of the lines were sown in the first decade of October. The experimental material was planted in single-row plots that were 1 m in length, with 30 seeds per row and 20 cm spacing between rows. The study was conducted over multiple growing seasons with two replications in a specialized infection nursery under artificial inoculation conditions. The complete set of wheat lines was grouped into compact replications, each subdivided into two contiguous blocks of 40 entries. Within each block, susceptible standard cultivars were systematically inserted after every 10 wheat lines. The nursery included five standard cultivars, consisting of four susceptible check cultivars (Almaly, Zhetysu, Steklovidnaya 24, and Bogarnaya 56) and one highly susceptible spreader cultivar (Morocco). In addition, a spreader row of the susceptible cultivar was established between the two replications to promote uniform airborne inoculum distribution and stable disease pressure throughout the experimental area. Border and end guard rows composed of susceptible standard cultivars were also planted around the nursery to promote uniform disease development and minimize edge effects (Figure S1). The experiment was established using a disease-screening nursery layout, which is commonly applied in wheat rust phenotyping to ensure homogeneous pathogen spread and disease development across a large number of genotypes [37,38]. The main agronomic practices for crop management were carried out in accordance with generally accepted methodological recommendations [39].

Meteorological parameters during the study period were recorded via an iMetos weather station (IMT300USW, Pessl Instruments, Weiz, Austria) at KRIAPG.

### 4.3. Screening for Resistance to Puccinia striiformis

The assessment and selection of winter wheat lines for resistance to yellow rust (*Puccinia striiformis* f. sp. *tritici*) were conducted under artificial infection conditions. The inoculation of the studied material was performed via a mixture of urediniospores of *Puccinia striiformis* and talc at a ratio of 1:100, with a spore load of 20 mg/m^2^ [40]. The population isolate of yellow rust used was collected from commercial wheat cultivars, breeding material, and wild grasses in the region.

The first disease assessment was carried out at the onset of symptom appearance, with subsequent evaluations performed at 7–10-day intervals until the milk–wax ripening stage of grain development. The evaluation of genotypes for resistance to yellow rust was based on infection type (IT) and disease severity (%). The infection type was determined via the following scale [41]: I (immune)—no visible symptoms; R (resistant)—small isolated necrotic lesions without pustules; MR (moderately resistant)—small pustules surrounded by chlorotic and necrotic areas; MS (moderately susceptible)—medium-sized pustules without necrosis, sometimes with chlorotic spots; and S (susceptible)—large pustules without chlorosis or necrosis.

Disease severity (0–100%) was assessed via the modified Cobb scale [42]. The infection coefficient (CI) for yellow rust was calculated by multiplying the constant values assigned to infection types by disease severity. The following constants were used: I = 0.0; R = 0.2; MR = 0.4; M = 0.6; MS = 0.8; and S = 1.0 [43].

### 4.4. Molecular Analyses

Genomic DNA was extracted from finely powdered seeds according to Dellaporta et al. with slight modifications [44,45]. PCR was performed in a 15 μL volume with 1 × PCR buffer, 1.5 mM MgCl_2_, 0.15 μM dNTPs, 0.7 μM primers, 0.5 U Taq DNA polymerase (GeneLab, Astana, Kazakhstan), and 3% DMSO. The reactions were carried out in a Bio-Rad iCycler system (Bio-Rad Laboratories, Hercules, CA, USA) with the following program: initial step at 95 °C for 4 min; 30 cycles at 94 °C for 1 min, 55–60 °C for 1 min, and 72 °C for 1 min; and a final step at 72 °C for 5 min. Separation and visualization of the DNA fragments (PCR products) were performed according to Babissekova et al. [46]. Briefly, the PCR products were analyzed via 8% polyacrylamide gel electrophoresis at a constant voltage of 200 V for 70 min and stained with ethidium bromide (Vilber, Lourmat, Collégien, France). Electrophoresis images were obtained via a Quantum ST4 gel-documenting system (Vilber, Lourmat, Collégien, France). Four DNA markers, including SSR and STS primers, were used to assess the yellow rust resistance of the hybrids (Table 6).

### 4.5. Statistical Analysis

All the statistical analyses, including analysis of variance (ANOVA), heatmaps and cluster analysis via the unweighted pair group method with arithmetic mean (UPGMA), were carried out in R (version 4.3.3) [51]. Post hoc pairwise comparisons were performed via Tukey’s HSD test to identify significant differences among means. In addition, descriptive statistics (means, medians, and interquartile ranges) were calculated to assess the variability of the studied traits. Cluster analysis was applied to identify genetically similar groups of lines and to differentiate them on the basis of their level of resistance.

## 5. Conclusions

Overall, the results indicate that the greatest breeding value lies in lines with low CI values (0–1), particularly those carrying combinations of *Yr5* and *Yr10*, whereas the predominance of MS forms highlights the need for further genetic improvement of the breeding material. Among the studied materials, only a limited number of lines presented high resistance to yellow rust (R, CI = 0–1). The most promising resistant lines included d.1010 (d.93 F3 (N23 × Kupava) × Mereke × 10/60 F5 N23 Kupava 7), Alpu/VR5053 (WA#FM/201/232/GS50A) × Steklovidnaya 24, Yr5/6* Avocet S × 20389-6, F5 N23 × Kupava/5 × 37/20948-8, MV-Menuett × 13/d 3 gene, SG-V9157 × 22/20060-3, and F5 N23 × Kupava/10 × Mamyr, all characterized by low infection coefficients (CI = 1). Several lines exhibited complete immunity to the pathogen (I, CI = 0), including Yr5/6* Avocet S × 16/6 × Triticum spelta; F5 N23 × Kupava/10 × 35/20060-2; Yr15/6* Avocet S × 20389-6; CH-111.14098 × OR2080111H; and Yr15/6* Avocet S × Sultan. The selected superior genotypes are recommended for use in breeding for resistance to yellow rust (*Puccinia striiformis* f. sp. *tritici*). The diversity of resistance genetic resources within the breeding material increases the immunological value of the developed cultivars.

## Figures and Tables

**Table 1:** Meteorological data for the study period.

Month	Air Temperature, °C					Precipitation, mm				
	2021	2022	2023	2024	2025	2021	2022	2023	2024	2025
March	4.1	5.8	8.4	5.4	6.2	117.9	168.6	61.2	135.5	76.8
April	12.4	16.7	11.9	12.8	15.7	56.3	46.8	68.2	111.3	57.2
May	19.4	19.0	17.2	17.6	20.8	81.6	145.4	43.4	121.2	80.4
June	23.1	24.3	24.6	24.5	25.6	20.9	35.9	4.3	19.7	17.1
July	26.9	26.5	27.1	25.0	27.7	22.8	15.1	33.6	85.2	9.6
Average	17.2	21.6	20.2	19.9	22.4	59.9	60.8	37.3	84.3	41.1

**Table 2:** Variability of the infection coefficient in wheat lines of the F2–F5 hybrid population (2024).

**F2**			**F3**		
n:30	Min.:	0.0	n:10	Min.:	0.00
	Median:	20.0		Median:	12.00
	Mean:	21.2		Mean:	11.80
	Max.:	50.0		Max.:	32.00
**F4**			**F5**		
n:21	Min.:	0.00	n:19	Min.:	0.000
	Median:	12.00		Median:	4.000
	Mean:	13.43		Mean:	7.947
	Max.:	50.00		Max.:	24.000

**Table 3:** Analysis of variance (ANOVA) of the coefficient of infection (CI).

Coefficient of Infection, CI					
	Df	Sum Sq	Mean Sq	F value	Pr (>F)
Hybrid	3	2250	750.1	6.172	0.000823 ***
Residuals	76	9236	121.5		

Signif. codes: *** *p* < 0.001.

**Table 4:** Multiple comparisons of the mean infection coefficient values in the wheat lines of the F2–F5 populations (Tukey’s HSD test).

Hybrid	Diff	Lwr	Upr	*p* Adj
F3-F2	−9.400000	−19.974078	1.1740784	0.0991130
F4-F2	−7.771429	−16.010684	0.4678269	0.0716281
F5-F2	−13.252632	−21.743141	−4.7621218	0.0005838
F4-F3	1.628571	−9.497564	12.7547072	0.9805298
F5-F3	−3.852632	−15.166089	7.4608260	0.8077021
F5-F4	−5.481203	−14.650092	3.6876858	0.4015702

**Table 5:** Size of the wheat hybrid population in 2025.

Type of Crossing	Year of Crossing	Generation	Lines
**Intraspecific hybridization** **(*Triticum aestivum* L.)** **pairwise—reciprocal crosses; complex—stepwise crosses**	2023	F_2_	30
	2022	F_3_	10
	2021	F_4_	21
	2020	F_5_	19

**Table 6:** Molecular markers and primers used for the identification of effective *Yr* resistance genes.

Gene	Marker Type	Primer Name	Primer Sequence	Amplicon Size (bp)	Reference
*Yr5*	STS	S19M93-100	TAATTGGGACCGAGAGACG TTCTTGCAGCTCCAAAACCT	100	Smith et al., 2007 [47]
		S23M41-275	TCAACGGAACCTCCAATTTC AGGTAGGTGTTCCAGCTTGC	275	
*Yr10*	SSR	Xpsp3000	GCAGACCTGTGTCATTGGTC GATATAGTGGCAGCAGCAGGATAC	260 (R) 240 (S)	Wang, L.F. et al., 2002 [48]
*Yr15*	SSR	Xbarc8	GCGGGAATCATGCATAGGAAAACAGAA GCGGG GCGAAACATACACATAAAAACA	250 (R) 280 (S)	Chen, X.M. et al., 1998 [49]
*Yr18*	STS	csLV34v	GTTGGTTAAGACTGGTGATGG TGCTTGCTATTGCTGAATAGT	150 (R) 229 (S)	Helguera, M. et al., 2003 [50]

## Data Availability

The original contributions presented in this study are included in the Supplementary Material. Further inquiries can be directed to the corresponding author.

## Supplementary Materials

The following supporting information can be downloaded at https://www.mdpi.com/article/10.3390/plants15131964/s1: Table S1: Assessment of resistance to yellow rust (*Puccinia striiformis* f. sp. *tritici*) in wheat lines under field conditions in southeastern Kazakhstan, 2023–2025. Table S2: Phenotypic and DNA marker-based screening of advanced wheat lines. Table S3: Lines of wheat hybrid populations (F2–F5). Table S4: Pedigree of the breeding lines. Figure S1: Schematic representation of the disease-screening nursery layout.

## Abbreviations

The following abbreviations are used in this manuscript:

IT	Infection type
CI	Coefficient of infection
ASR	All-stage resistance
APR	Adult plant resistance
STS	Sequence Tagged Site
SSR	Simple Sequence Repeat
KRIAPG	Kazakh Research Institute of Agriculture and Plant Growing
CIMMYT	International Maize and Wheat Improvement Center
