# Supplementary material for: Genetic Diversity and Breeding Strategies for Resistance to Yellow Rust (Puccinia striiformis f. sp. tritici) in Wheat Hybrid Populations Based on Phenotypic and DNA Marker Screening
Source: Plants (Basel). 2026 Jun 25;15(13):1964. doi: 10.3390/plants15131964 (PMC13364376; doi:10.3390/plants15131964)
Supplement: Supplementary file 1 [file plants-15-01964-s001.zip › Figure S1.pdf]

Figure S1. Schematic representation of the disease-screening nursery layout.

BORDER GUARD ROW (SC)

REPLICATION I

Block 1 (40 entries)

L1–10 | SC | SR | L11–20 | SC | SR | L21–30 | SC | SR | L31–40 | SC | SR

Block 2 (40 entries)

L41–50 | SC | SR | L51–60 | SC | SR | L61–70 | SC | SR | L71–80 | SC | SR

SR (Morocco)
Central spreader row

REPLICATION II

Block 1 (40 entries)

L1–10 | SC | SR | L11–20 | SC | SR | L21–30 | SC | SR | L31–40 | SC | SR

Block 2 (40 entries)

L41–50 | SC | SR | L51–60 | SC | SR | L61–70 | SC | SR | L71–80 | SC | SR

BORDER GUARD ROW (SC)

L = wheat breeding lines

SC = susceptible check cultivars

SR = susceptible spreader row
