# Supplementary material for: Genetic Diversity and Breeding Strategies for Resistance to Yellow Rust (Puccinia striiformis f. sp. tritici) in Wheat Hybrid Populations Based on Phenotypic and DNA Marker Screening
Source: Plants (Basel). 2026 Jun 25;15(13):1964. doi: 10.3390/plants15131964 (PMC13364376; doi:10.3390/plants15131964)
Supplement: Supplementary file 1 [file plants-15-01964-s001.zip › Table S1.pdf]

Table S1 Assessment of resistance to yellow rust (*Puccinia striiformis* f. sp. *tritici*) in wheat lines under field conditions in southeastern Kazakhstan, 2023–2025

Entry	Lines	*Puccinia striiformis* f. sp. *tritici*		
		IT*	DS, %**	CI***
1	18723-7 x Art x Saulesku#44/TR81020/3/Agri/Nac//Kauz	MS-S	5-30	30
2	Almaly x Krasnovodopadskaya-25//PBW343*2/Kukuna x Fielder	MS-S	10-30	30
3	Steklovidnaya 24 x Moskovskaya 56 x Vilmorin 23	MR-MS	10-25	20
4	Mamyr x Bonito-44 x Yr7/ 6* Avocet S	MS-S	10-40	40
5	Karasai x Moskovskaya 56 x Clement	MR-MS	15-20	16
6	Mamyr x Bonito-44 x Anza	MR-MS	10-30	24
7	Yr5/ 6* Avocet S x 16/12 x Triticum spelta	I	0	0
8	Yr15/ 6* Avocet S x 13/d 3 gen x Yr15/ 6* Avocet S	MS	5-15	12
9	9/7/128 gen x Yr17/6*Avocet S	MS	10-20	16
10	9/7/128 gen x Ajvina	MR-MS	10	4
11	4/2109 x Yr5/ 6* Avocet S	R-MR	5-10	4
12	Mamyr x Bonito-44	MS-S	30-50	50
13	6/124 (gen) x Ajvina	MS	10-20	16
14	7/19251-2 x Saulesku#44/TR81020/3/Agri/Nac//Kauz	MS	5-20	16
15	18723-7 x Ajvina	MS	5-20	16
16	27/20156-3 x Saulesku#44/TR81020/3/Agri/Nac//Kauz	MS-S	20-30	30
17	Yr5/ 6* Avocet S x 16/12	MS-S	20-40	40
18	20841-17 x Batera//Kea/Tow/3/Tam200/4/494J6.11/Trap#1/Bow/5 /TX96	MS	10-20	16
19	Egemen 20 x Adagio	MS	15-25	20
20	YrSP / 6* Avocet S x16/12	MS	5-10	8
21	Yr15/ 6* Avocet S x 13/ d 3 gen	MS	10-20	16
22	Almaly x Krasnovodopadskaya-25//PBW343*2/Kukuna	MS	15-25	20
23	Steklovidnaya 24 x Moskovskaya 56	MS	15-30	24
24	Karasai x Moskovskaya 56	MS	15-25	20
25	Steklovidnaya 24 x Adagio	MS	20-40	32
26	18723-7 x Art	MS	10-25	20
27	4/19059-21 x Seri	MS	20-50	40
28	20389-3 x Batera//Kea/Tow/3/Tam200/4/ 494J6.11/Trap#1/Bow/5/TX96V2427	MS	15-25	20
29	19059-21 x Krasnovodopadskaya-25//PBW343*2/Kukuna	MS	10-25	20
30	9/20197-17 x YrSP / 6* Avocet S	MS	10-20	16
31	F5 N23 x Kupava /10 x 35/20060-2	I	0	0
32	d.1010(d.93 F3(N23 x Kupava) x Mereke x 10/60 F5 N23 Kupava 7	R	5	1
33	SO1-249-3*R x 7/19251-2	MS	5	4
34	20841-17 x Ilinca	MS	20-30	24
35	Moskovskaya 56 x 32/20232-14	MS	10-15	12
36	19051-11 x SO1-249-3*R	MS	10-20	16
37	20388-3 x Dh-Lines 1-1	MS	15-20	16
38	19670-1 x SO1-249-3*R	MS	5-15	12
39	Saulesku#44/TR81020/3/Agri/Nac//Kauz x Dimash	MS	25-40	32
40	Alpu/VR5053(WA#FM/201/23*2/GS50A) x Steklovidnaya 24	R	5	1
41	Clement x Egemen 20	MS	5-15	12
42	Yr15/ 6* Avocet S x 20389-6	I	0	0
43	Yr5/ 6* Avocet S x 20389-6	R	5	1
44	Yr5/ 6* Avocet S x SWW 1/904	MS	10-25	20

45	YrSP / 6* Avocet S x Steklovidnaya 24	MS	25-40	32
46	F5 N23 x Kupava /5 x 37/20948-8	R	5	1
47	DI09016 x 5/126 gen	MS	20-30	24
48	Fulvio x Daulet	MS	5-15	12
49	Yr10/ 6* Avocet S x 38/20389-3	MR-MS	5	4
50	Mv Zelma x 18952-1	MS	15-25	20
51	Subtil x Almaly	MR-MS	10	8
52	Subtil x Dinara	MS	5-10	8
53	Lia 5823-8 x 4/2109	MS	5	4
54	Mv-Menuett x 13/ d 3 gen	R	5	1
55	Tres/6* AVS x 19187-3	S	30-50	50
56	Yr15/ 6* Avocet S x Mereke 70	MS	10-20	16
57	Lia 5899-16 x 9/7/128 gen	MS	5-20	16
58	Mv-Menuett x 18952-1	MS	10-15	12
59	Beavborg x Arap	MS	20-25	20
60	Beavborg x 20156-4	MS	15-25	20
61	Beavborg x 5/126 gen	R	5	1
62	F5 N23 x Kupava /1 x 4/19059-21	MS	20-30	24
63	F5 N23 x Kupava /1 x 23/20061-12	MS	15-25	20
64	SG-V9157 x 22/20060-3	R	5	1
65	SWW1-135 x F2 hybr.lab. (F5 N23 x Kupava /1 x 48/12121-6)	MS	20-30	24
66	SG-V9157 x 23/20061-12	R-MR	5-10	4
67	CH-111.14098 x OR2080111H	I	0	0
68	Dh-Lines 1-1 x KS940786-6-9FM/CO970547-7	MR	10	4
69	Dh-Lines 1-1 x 15/280 gen	MS	20-30	24
70	Yr10/ 6* Avocet S x Sultan - 2	MS	10-20	16
71	Yr15/ 6* Avocet S x Sultan	I	0	0
72	BC01131-24 x Avicenna	R	5	1
73	F5 N23 x Kupava /3 x Nureke	R-MR	5-15	6
74	F5 N23 x Kupava /10 x Mamyr	R	5	1
75	F5 N23 x Kupava /1 x 48/12121-6	MR	15	6
76	Erythrospermum 1290-08 x 19030-1	R	5	1
77	SO1-249-14*R x 57/21190-1	MR	10-20	8
78	CH111.14511 x 13/10210	MR	10	4
79	SG-V9157 x 18723-7	MR	10-15	6
80	Dh-Lines 1-1 x 20153-2	R	5	1
St. Almaly		MS-S	10-30	30
St. Zhetysu		MS	10-30	24
St. Steklovidnaya 24		MS	5-40	32
St. Bogarnaya 56		MS-S	20-60	60
St. Morocco		S	80-100	100

* IT – infection type (I – immune, R – resistant, MR – moderately resistant, MS – moderately susceptible, S – susceptible), **DS – disease severity (%), ***CI – coefficient of infection
