# Supplementary material for: Genetic Diversity and Breeding Strategies for Resistance to Yellow Rust (Puccinia striiformis f. sp. tritici) in Wheat Hybrid Populations Based on Phenotypic and DNA Marker Screening
Source: Plants (Basel). 2026 Jun 25;15(13):1964. doi: 10.3390/plants15131964 (PMC13364376; doi:10.3390/plants15131964)
Supplement: Supplementary file 1 [file plants-15-01964-s001.zip › Table S2.pdf]

Table S2 Phenotypic and DNA marker–based screening of advanced wheat lines

Entry	Lines	*Yr5*	*Yr10*	*Yr15*	*Yr18*	Resistance level		CI***
						IT*	%**	
1	4/2109 x Yr5/ 6* Avocet S	+	-	-	heterozygous (+/–)	MR	10	4
2	Yr15/ 6* Avocet S x 13/ d 3 gen	+	-	heterozygous (+/–)	+	MS	20	16
3	Yr15/ 6* Avocet S x 13/ d 3 gen x Yr15/ 6* Avocet S	+	-	heterozygous (+/–)	+	MS	15	12
4	Karasai x Moskovskaya 56 x Clement	+	-	-	heterozygous (+/–)	MS	20	16
5	Yr5/ 6* Avocet S x 16/12 x Triticum spelta	+	-	-	heterozygous (+/–)	I	0	0
6	F5 N 23 x Kupava /10 x 35/20060-2	+	-	-	-	I	0	0
7	d.1010(d.93 F3(N23 x Kupava) x Mereke x 10/60 F5 N23 Kupava 7	+	-	-	heterozygous (+/–)	R	5	1
8	SO1-249-3*R x 7/19251-2	-	-	-	-	MS	5	4
9	Moskovskaya 56 x 32/20232-14	+	-	-	-	MS	15	12
10	19670-1 x SO1-249-3*R	-	-	-	-	MS	15	12
11	Alpu/VR5053(WA#FM/201/23*2/GS50A) x Steklovidnaya 24	+	+	-	heterozygous (+/–)	R	5	1
12	Yr15/ 6* Avocet S x 20389-6	+	-	-	-	I	0	0
13	Yr5/ 6* Avocet S x 20389-6	+	+	-	-	R	5	1
14	Yr5/ 6* Avocet S x SWW 1/904	+	-	-	heterozygous (+/–)	MS	25	20
15	YrSP / 6* Avocet S x Steklovidnaya 24	+	+	-	-	MS	40	32
16	Yr10/ 6* Avocet S x 38/20389-3	-	+	-	-	MS	5	4
17	Subtil x Almaly	+	+	-	-	MS	10	8
18	F5 N 23 x Kupava /5 x 37/20948-8	+	+	-	heterozygous (+/–)	R	5	1
19	MV-Menuett x 13/ d 3 gen	+	-	-		R	5	1
20	Yr15/ 6* Avocet S x Mereke 70	-	-	-	heterozygous (+/–)	MS	20	16
21	F5 N23 x Kupava /1 x 4/19059-21	-	-	-	-	MS	30	24
22	F5 N23 x Kupava /1 x 23/20061-12	+	-	-	-	MS	25	20
23	SG-V9157 x 22/20060-3	+	+	-	-	R	5	1
24	CH-111.14098 x OR2080111H	-	+	-	-	I	0	0
25	Yr10/ 6* Avocet S x Sultan - 2	-	-	+	+	MS	20	16
26	Yr15/ 6* Avocet S x Sultan	+	-	-	+	I	0	0
27	SWW1-135 x F2 hybr.lab. (F5 N23 x Kupava /1 x 48/12121-6)	+	-		+	MS	30	24
28	F5 N23 x Kupava /10 x Mamyr	+	-	-	-	R	5	1
29	F5 N23 x Kupava /1 x 48/12121-6	+	-	-	-	MR	15	6
30	SO1-249-14*R x 57/21190-1	+	-	-	-	MR	20	8
31	DH-Lines 1-1 x 20153-2	+	-	-	-	R	5	1
32	*Yr5*	+						
33	*Yr10*		+					
34	*Yr15*			+				
35	*Yr18*				+			

*IT – infection type (I – immune, R – resistant, MR – moderately resistant, MS – moderately susceptible), **DS – disease severity (%), ***CI – coefficient of infection
