# Supplementary material for: Genetic Diversity and Breeding Strategies for Resistance to Yellow Rust (Puccinia striiformis f. sp. tritici) in Wheat Hybrid Populations Based on Phenotypic and DNA Marker Screening
Source: Plants (Basel). 2026 Jun 25;15(13):1964. doi: 10.3390/plants15131964 (PMC13364376; doi:10.3390/plants15131964)
Supplement: Supplementary file 1 [file plants-15-01964-s001.zip › Table S3.pdf]

Table S3 Lines of wheat hybrid populations ($F_2 - F_5$)

Entry	Lines
	F_2
1	18723-7/ARTxSaulesku#44/TR81020/3/Agri/Nac//Kauz
2	Almaly/Krasnovodopadskaya-25//PBW343*2/Kukuna x Fielder
3	Steklovidnaya 24/Moskovskaya 56/Vilmorin 23
4	Mamyr/Bonito-44/Yr7/ 6* Avocet S
5	Karasai/Moskovskaya 56/Clement
6	Mamyr/Bonito-44/Anza
7	Yr5/ 6* Avocet S/16/12/Triticum spelta
8	Yr15/ 6* Avocet S/13/d 3 gen/Yr15/ 6* Avocet S
9	9/7/128 gen/YR17/6*Avocet S
10	9/7/128 gen/Ajvina
11	4/2109/Yr5/ 6* Avocet S
12	Mamyr/Bonito-44
13	6/124 (gen)/Ajvina
14	7/19251-2/Saulesku#44/TR81020/3/Agri/Nac//Kauz
15	18723-7/Ajvina
16	27/20156-3/Saulesku#44/TR81020/3/Agri/Nac//Kauz
17	Yr5/ 6* Avocet S/16/12
18	20841-17/Batera//Kea/Tow/3/Tam200/4/494J6.11/Trap#1/Bow/5/TX96
19	Egemen 20/Adagio
20	YrSP / 6* Avocet S/16/12
21	Yr15/ 6* Avocet S/13/d 3 gen
22	Almaly/Krasnovodopadskaya-25//PBW343*2/Kukuna
23	Steklovidnaya 24/Moskovskaya 56
24	Karasai/Moskovskaya 56
25	Steklovidnaya 24/Adagio
26	18723-7/Art
27	4/19059-21/Seri
28	20389-3/Batera//Kea/Tow/3/Tam200/4/ 494J6.11/Trap#1/Bow/5/TX96V2427
29	19059-21/Krasnovodopadskaya-25//PBW343*2/Kukuna
30	9/20197-17/YrSP / 6* Avocet S
	F_3
31	F5 N23/Kupava /10/35/20060-2
32	d.1010(d.93 F3(N23/Kupava)/Mereke/10/60 F5 N23 Kupava 7
33	SO1-249-3*R/7/19251-2
34	20841-17/Ilinca
35	Moskovskaya 56/32/20232-14
36	19051-11/SO1-249-3*R
37	20388-3/Dh-Lines 1-1
38	19670-1/SO1-249-3*R
39	Saulesku#44/TR81020/3/Agri/Nac//Kauz/Dimash
40	Alpu/VR5053(WA#FM/201/23*2/GS50A)/Steklovidnaya 24
	F_4
41	Clement/Egemen 20
42	Yr15/ 6* Avocet S/20389-6
43	Yr5/ 6* Avocet S/20389-6
44	Yr5/ 6* Avocet S/SWW 1/904
45	YrSP / 6* Avocet S/Steklovidnaya 24
46	F5 N23 x Kupava /5/ 37/20948-8
47	DI09016/5/126 gen
48	Fulvio/Daulet
49	Yr10/ 6* Avocet S/38/20389-3
50	Mv Zelma/18952-1
51	Subtil/Almaly
52	Subtil/Dinara

53	Lia 5823-8/4/2109
54	Mv-Menuett/13/ d 3 gen
55	Tres/6* AVS/19187-3
56	Yr 15/ 6* Avocet S/Mereke 70
57	Lia 5899-16/9/7/128 gen
58	Mv-Menuett/18952-1
59	Beavborg/Arap
60	Beavborg/20156-4
61	Beavborg/5/126 gen
	F_5
62	F5 N23 x Kupava /1/ 4/19059-21
63	F5 N23 x Kupava /1/23/20061-12
64	SG-V9157/22/20060-3
65	SWW1-135/F2 hybr.lab. (F5 N23/Kupava /1/48/12121-6)
66	SG-V9157/23/20061-12
67	CH-111.14098/OR2080111H
68	Dh-Lines 1-1/KS940786-6-9FM/CO970547-7
69	Dh-Lines 1-1/15/280 gen
70	Yr10/ 6* Avocet S/Sultan - 2
71	Yr15/ 6* Avocet S/Sultan
72	BC01131-24/Avicenna
73	F5 N23/Kupava /3/Nureke
74	F5 N23/Kupava /10/Mamyr
75	F5 N23/Kupava /1/48/12121-6
76	Erythrospermum 1290-08/19030-1
77	SO1-249-14*R/57/21190-1
78	CH111.14511/13/10210
79	SG-V9157/18723-7
80	Dh-Lines 1-1/20153-2

St. Almaly
St. Zhetysu
St. Steklovidnaya 24
St. Bogarnaya 56
St. Morocco
