# Supplementary material for: Genetic Diversity and Breeding Strategies for Resistance to Yellow Rust (Puccinia striiformis f. sp. tritici) in Wheat Hybrid Populations Based on Phenotypic and DNA Marker Screening
Source: Plants (Basel). 2026 Jun 25;15(13):1964. doi: 10.3390/plants15131964 (PMC13364376; doi:10.3390/plants15131964)
Supplement: Supplementary file 1 [file plants-15-01964-s001.zip › Table S4.pdf]

Table S4 Pedigree of the breeding lines

Lines	Pedigree
18723-7	Rasad x Salamon
7/19251-2	Er 8794 x Naz
27/20156-3	MK 3677 x Karasai
20841-17	Oktava x Zhalyn
20389-3	Zhalyn x Naz
19059-21	Adyr x Kiyal
9/20197-17	Kin-4 x Almaly
35/20060-2	Im-78 x Steklovidnaya 24
32/20232-14	Naz x Delta
9/7/128 gen	F5N23 Kupava /3
19051-11	Reke x Naz
20388-3	Naz x d15 KSI bogara 07
19670-1	19544 x Naz
20389-6	Zhalyn x Naz
5/126 gen	F2N23 Kupava
19187-3	Kharkovskaya 107 x Steklovidnaya 24
20156-4	MK 3677 x Karasai
5/126 gen	F2 N23 Kupava
4/19059-21	Adyr x Kiyal
23/20061-12	Im-78 x Karasai
22/20060-3	Im-78 x Steklovidnaya 24
4/2109	F5N23 Kupava /7
48/12121-6	Arap x Kyzylbidai
15/280 gen	F130L-1-12 x MV12(Atill-12)
19030-1	Yuzhnaya 12 x Naz
57/21190-1	20982 x Akbiday
20153-2	Tircos 215 x Aliya
